# Prevalence of cognitive impairment and its related factors among Chinese older adults: an analysis based on the 2018 CHARLS data

**DOI:** 10.3389/fpubh.2024.1500172

**Published:** 2024-12-24

**Authors:** Xueqin Wu, Yufu Tang, Yushan He, Qiwei Wang, Yinhui Wang, Xiujun Qin

**Affiliations:** ^1^School of Public Health, Shanxi Medical University, Taiyuan, China; ^2^Shanxi Provincial Key Laboratory of Drug Toxicology and Radiation Damage Drugs, Department of Radiology and Environmental Medicine, China Institute for Radiation Protection, Taiyuan, China; ^3^Division of Radiology and Environmental Medicine, China Institute for Radiation Protection, Taiyuan, China; ^4^School of Pharmacy, Tongji Medical College, Huazhong University of Science and Technology, Wuhan, China

**Keywords:** cognitive impairment, Chinese older adult population, risk factors, prevalence, CHARLS

## Abstract

**Background:**

Cognitive impairment is a major public health concern in aging societies. This study aimed to investigate the prevalence of cognitive impairment and its associated factors among Chinese adults aged 60 years and older using data from the 2018 China Health and Retirement Longitudinal Study (CHARLS).

**Methods:**

Utilizing data from the 2018 wave of CHARLS, we assessed participants’ cognitive status using the Mini-Mental State Examination (MMSE), and the influencing factors related to cognitive impairment were analyzed by using the chi-square test and multifactor logistic regression. The prevalence of cognitive impairment was stratified by gender, education level, residence, marital status, daytime napping, and nighttime sleep duration, and the trend of cognitive impairment prevalence with age was observed.

**Results:**

9,804 participants were finally included in the study and the overall prevalence of cognitive impairment was 44.04% (95%CI, 43.02–45.06%). The prevalence was significantly higher in females (50.8%) than males (37.1%), and increased with age, from 41.5% in those aged 60–64 years to 57.7% in those aged ≥75 years. Lower educational level, rural residence, and being divorced/ widowed/unmarried were associated with a higher prevalence of cognitive impairment (all *p* < 0.001). Multifactor logistic regression indicated that older age (OR = 1.51, 95%CI, 1.33–1.73 for ≥75 vs. 60–64 years), female gender (OR = 1.54, 95%CI, 1.35–1.77), higher education (OR = 0.46, 95%CI, 0.42–0.51 for primary school and below vs. illiteracy), rural areas (OR = 2.35, 95%CI, 2.07–2.65 for village vs. the center of city/town), divorced/ widowed/unmarried status (OR = 1.40, 95%CI, 1.25–1.57) and participation in physical activity (OR = 0.80, 95%CI, 0.73–0.87) were significantly associated with cognitive impairment.

**Conclusion:**

Cognitive impairment is highly prevalent among older adults in China with substantial demographic disparities. Targeted interventions and public health strategies are needed to promote cognitive health in this rapidly aging population.

## 1 Introduction

Cognitive impairment, characterized by deficits in one or more cognitive domains such as memory, attention, language, and executive function, is a major public health concern in aging societies worldwide. It represents a continuum of cognitive decline, with mild cognitive impairment (MCI) marking the intermediate stage between normal cognitive aging and dementia (1). If MCI progresses, it can lead to dementia, resulting in a substantial loss of cognitive abilities and the capacity for independent living. Individuals with MCI are at a significantly increased risk of progressing to dementia, with a conversion rate as high as 15% (2). Qiu Y, et al. (3) conducted a systematic evaluation and meta-analysis of cognitive decline in community-dwelling older adults showing that the prevalence of cognitive decline has been increasing in recent years. As the global population ages, the prevalence of cognitive impairment and dementia is projected to rise sharply in the coming decades, posing enormous challenges to healthcare systems, social services, and economies (4). It has been predicted that by 2050, the number of people with dementia will reach 115.4 million, with 71% of dementia patients living in low- or middle-income countries (5). China, the world’s most populous country, is experiencing an unprecedented pace of population aging. In 2021, China’s population aged 65 and over to approach 200 million, with projections estimating that this number will rise to 395 million by 2050 (6). The rapid aging of the Chinese population has profound implications for the country’s health and social welfare systems, as age is the strongest known risk factor for cognitive impairment and dementia (7). Despite the growing recognition of cognitive impairment as a public health priority, epidemiological data on its prevalence and risk factors in the Chinese population remain limited and inconsistent. Previous studies have reported prevalence estimates ranging from 15.4 to 26.48% in Chinese adults aged ≥60 years (8–10), with differences in the prevalence of cognitive impairment likely due to differences in diagnostic criteria, assessment methods, and sample characteristics. In this study we analyzed data from the 2018 wave of the China Health and Retirement Longitudinal Study (CHARLS), a nationally representative cross-sectional survey of residents aged ≥45 years in China, we aimed to assess the prevalence of cognitive impairment and its major sociodemographic and behavioral-related factors in Chinese older adults, in order to provide relevant scientific evidence for the prevention of further development and deterioration of cognitive impairment and to reduce the dementia disease.

## 2 Materials and methods

### 2.1 Data source and study population

**Figure 1:**
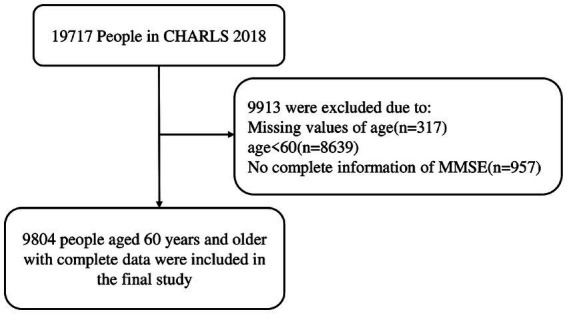
Flow chart for enrollment of study participants.

The data for this study were obtained from the China Health and Retirement Longitudinal Study (CHARLS) database, an ongoing nationwide longitudinal research program. CHARLS has been conducting nationwide baseline surveys since 2011, with follow-up surveys in 2013, 2015, 2018, and 2020, covering 150 counties and 450 communities (villages) of 28 provinces (autonomous regions and municipalities), using a multi-stage probability-proportional-to-size random sampling method to obtain samples at both the county/district and village sampling stages to ensure that the samples were comprehensively representative. The CHARLS data were collected through one-on-one interviews using a standardized questionnaire that covered information on demographics, lifestyle, and health status (11). Due to the impact of the COVID-19 pandemic in 2020, residents’ lifestyles have changed considerably (12, 13), and the relevant survey factors may have different degrees of bias, so our study chose the fourth wave of data from 2018 as the cross-sectional study data. Our study was approved by the Biomedical Ethics Committee of Peking University under the approval number IRB00001052-11015. All participants provided written informed consent during the survey. The inclusion criteria for this study were (1) those aged 60 years and above and (2) those with information on cognitive function assessment. Exclusion criteria were (1) age less than 60 years, (2) lack of or imperfect information on cognitive function assessment. The overall study design flowchart is shown in Figure 1.

### 2.2 Assessment of cognitive function

Cognitive function was assessed using the Chinese version of the MMSE, and its good validity and reliability have been proven among Chinese older adults (14, 15). The assessment scale consists of 30 items with a total score range of 0–30, covering four dimensions: orientation, attention, situational memory, and visuospatial ability. Orientation was assessed by asking participants about the year, date, season, and location; attention was assessed by asking participants to perform an arithmetic problem in which they subtracted 100 from 7 five times in a row; situational memory was measured by immediate and delayed word recall; and visuospatial ability was assessed by picture recognition and drawing, with one point awarded for a correct answer to each item, with higher scores indicating better cognitive functioning, and grouping according to educational level (16), with a score of <17 for illiteracy, <20 for elementary school, and < 24 for middle school and above being considered to have cognitive dysfunction, otherwise, it is considered to be the absence of cognitive dysfunction (17).

### 2.3 Data analysis

**Figure 2:**
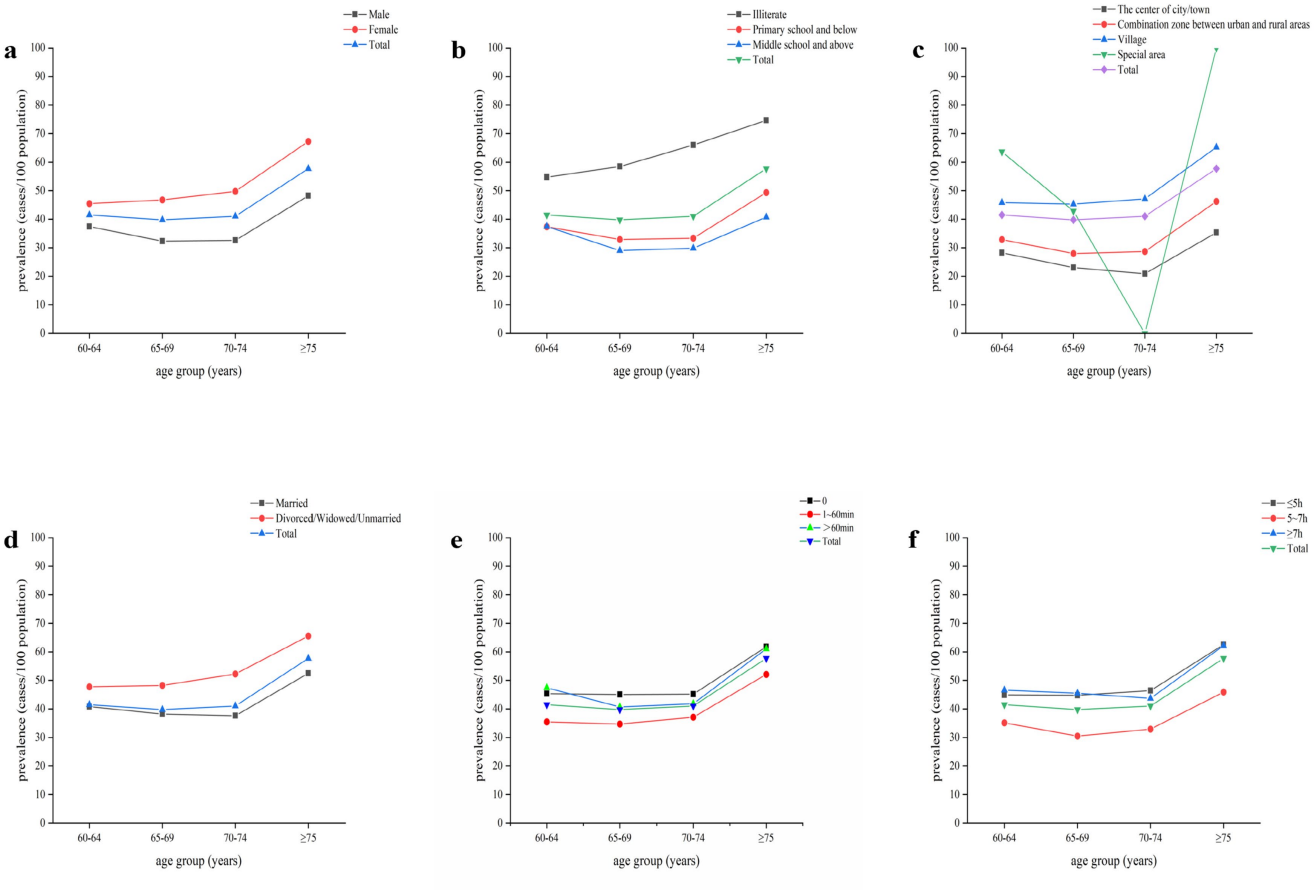
Age trends in the prevalence of cognitive impairment. **(a)** stratified by gender. **(b)** stratified by education level. **(c)** stratified by residence. **(d)** stratified by marital status. **(e)** stratified by daytime napping. **(f)** stratified by nighttime sleep duration.

Data cleaning and merging of CHARLS 2018 cross-sectional data were performed with Stata18.0, and SPSS27.0 was used to perform statistical tests. Firstly, descriptive statistical analysis was performed for socio-demographic characteristic factors, health-related factors, and lifestyle factors, in which the measurements that conformed to normal distribution were expressed as mean and standard deviation, whereas those not conforming to normal distribution were expressed as median and interquartile spacing [M (P25, P75)]. In this study, cognitive function scores were continuous variables, and the others were categorical variables; information on categorical variables was presented as frequencies and percentages (*n*, %), and statistical tests were performed using the chi-square test or Fisher’s exact test. Secondly, statistically significant variables in the one-way analysis were integrated into the multifactorial logistic regression model using a step-forward approach to explore the influencing factors associated with cognitive impairment. *p* < 0.05 was considered statistically significant (Figure 2).

## 3 Results

### 3.1 Characteristics of the study population

Of the 19,717 individuals who participated in the 2018 wave of CHARLS, 9,804 (49.7%) were included in the present analysis after excluding those missing data on age (*n* = 8,956) or cognitive function assessment (*n* = 957). The mean age of the included participants was 68.68 [Standard Deviation (SD) = 6.568], 49.4% were male and 50.6% were female. The average MMSE score was 19.75[Standard Deviation (SD) = 6.302]. The majority of participants had a primary school education or below (74.3%), were married (80.1%), and resided in rural areas (73.4%). Table 1 shows the characteristics of the study population by cognitive status.

**Table 1:** General characteristics and prevalence of cognitive impairment in the study population.

Variables	*N*	%	Cognitive impairment (*n*)	Prevalence of cognitive impairment (%)
Gender				
Male	4,845	49.4	1799	37.1
Female	4,959	50.6	2,519	50.8
Age				
60 ~ 64	3,148	32.1	1,307	41.5
65 ~ 69	2,967	30.3	1,180	39.8
70 ~ 74	1790	18.3	735	41.1
≥75	1899	19.4	1,096	57.7
Education level				
Illiterate	2,852	29.1	1797	63.0
Primary school and below	4,429	45.2	1,642	37.1
Middle school and above	2,523	25.7	879	34.8
Residence				
The center of city/town	1934	19.7	522	27.0
combined urban and rural areas	651	6.6	213	32.7
Village	7,193	73.4	3,570	49.6
Special area	26	0.3	13	50.0
Marital status				
Married	7,855	80.1	3,231	41.1
Divorced/Widowed/Unmarried	1949	19.9	1,087	55.8
Chronic disease				
Yes	4,628	47.2	2029	43.8
No	5,176	52.8	2,289	44.2
Daytime napping				
1 ~ 60 min	4,101	41.8	1,585	38.6
>60 min	2011	20.5	948	47.1
0	3,692	37.7	1785	48.3
Nighttime sleep duration				
≤5 h	3,576	36.5	1753	49.0
5 ~ 7 h	3,467	35.4	1,213	35.0
≥7 h	2,761	28.2	1,352	49.0
Social communication				
Yes	4,790	48.9	1788	37.3
No	5,014	51.1	2,530	50.5
Intensive physical activity				
Yes	2,644	27.0	1,201	45.4
No	7,160	73.0	3,117	43.5
Moderate physical activity				
Yes	4,380	44.7	1727	39.4
No	5,424	55.3	2,591	47.8
Light physical activity				
Yes	7,994	81.5	3,356	42.0
No	1810	18.5	962	53.1
Smoking				
Yes	4,376	44.6	1725	39.4
No	5,428	55.4	2,593	47.8
Drinking				
Yes	3,135	32.0	1,140	36.4
No	6669	68.0	3,178	47.7

### 3.2 Prevalence of cognitive impairment

The overall prevalence of cognitive impairment among Chinese adults aged ≥60 years was 44.04% (95% CI, 43.02–45.06%). Women had a significantly higher prevalence than men (50.8% vs. 37.1%; *p* < 0.001). The prevalence of cognitive impairment increased with age, from 41.5% in those aged 60–64 years to 57.7% in those aged ≥75 years (P for trend <0.001). Participants with lower educational levels had higher prevalence rates, which were 63.0, 37.1, and 34.8% for those who were illiterate, had primary school education or below, and had middle school education or above, respectively (*p* < 0.001). The prevalence of cognitive impairment also varied significantly by marital status, residence, daytime napping, nighttime sleep duration, social activities, physical activities, smoking, and alcohol consumption (all *p* < 0.05) (Table 1).

### 3.3 Factors associated with cognitive impairment

Table 2 presents the results of the univariable analysis of factors associated with cognitive impairment. Older age, female gender, lower education, rural residence, being widowed/divorced/unmarried, lack of daytime napping, short or long nighttime sleep duration, lack of social activities, lack of light or moderate physical activity, and no history of smoking or alcohol consumption were significantly associated with a higher prevalence of cognitive impairment (all *p* < 0.05), presenting a chronic disease and heavy physical activity showed no significant difference in the prevalence of cognitive impairment(*p* > 0.05). The Hosmer-Lemeshow test, yielding a *p*-value of 0.096, indicated a good model fit for the multifactor logistic regression analysis. The analysis revealed that significant risk factors for cognitive impairment: older age (OR = 1.51, 95%CI, 1.33–1.73 for ≥75 vs. 60–64 years), female gender (OR = 1.54, 95%CI, 1.35–1.77), rural residence (OR = 2.35, 95%CI, 2.07–2.65), divorced/widowed/unmarried status (OR = 1.40, 95%CI, 1.25–1.57) and people with a history of smoking (OR = 1.13, 95% CI, 1.00–1.29).Protective factors for the development of cognitive impairment: higher education (OR = 0.46, 95%CI, 0.42–0.51 for primary school and below vs. illiteracy), compared to no daytime napping, daytime napping ranged from 1 to 60 min (OR = 0.80, 95%CI, 0.72–0.88), compared to sleep duration <5 h, nighttime sleep duration 5-7 h (OR = 0.69, 95%CI, 0.62–0.76), participation in social activities(OR = 0.70, 95%CI, 0.64–0.76), moderate physical activity (OR = 0.80, 95%CI, 0.73–0.87), light physical activity (OR = 0.84, 95%CI, 0.75–0.94), and a history of alcohol consumption (OR = 0.87, 95% CI, 0.79–0.97). As shown in Table 3.

**Table 2:** One-way analysis of independent variables for cognitive impairment.

Variables	Cognitive normal (*n*, %)	Cognitive impairment (*n*, %)	t/χ^2^	*p*
Demographic backgrounds				
Gender			185.696**	<0.001
Male	3,046 (55.52)	1,799 (41.66)		
Female	2,440 (44.48)	2,519 (58.34)		
Age			180.593**	<0.001
60 ~ 64	1,841 (33.56)	1,307 (30.27)		
65 ~ 69	1,787 (32.57)	1,180 (27.33)		
70 ~ 74	1,055 (19.23)	735 (17.02)		
≥75	803 (14.64)	1,096 (25.38)		
Education level			590.238**	<0.001
Illiterate	1,055 (19.23)	1,797 (41.62)		
Primary school and below	2,787 (50.80)	1,642 (38.03)		
Middle school and above	1,644 (29.97)	879 (20.36)		
Residence			353.590**	<0.001
The center of city/town	1,412 (25.74)	522 (12.09)		
combined urban and rural areas	438 (7.98)	213 (4.93)		
Village	3,623 (66.04)	3,570 (82.68)		
Special area	13 (0.24)	13 (0.30)		
Marital status			135.786**	<0.001
Married	4,624 (84.29)	3,231 (74.83)		
Divorced/Widowed/Unmarried	862 (15.71)	1,087 (25.17)		
Health status				
Chronic disease			0.114	0.704
Yes	2,599 (47.38)	2,029 (46.99)		
No	2,887 (52.62)	2,289 (53.01)		
Daytime napping			84.004**	<0.001
1 ~ 60 min	2,516 (45.86)	1,585(36.71)		
>60 min	1,063 (19.38)	948(21.95)		
0	1907 (34.76)	1,785(41.34)		
Nighttime sleep duration			178.501**	<0.001
≤5 h	1823 (33.23)	1,753 (40.60)		
5 ~ 7 h	2,254 (41.09)	1,213 (28.09)		
≥7 h	1,409 (25.68)	1,352 (31.31)		
Living/Lifestyle				
Social communication			171.387**	<0.001
Yes	3,002 (54.72)	1,788 (41.41)		
No	2,484 (45.28)	2,530 (58.59)		
Intensive physical activity			2.799	0.094
Yes	1,443 (26.30)	1,201 (27.81)		
No	4,043 (73.70)	3,117 (72.19)		
Moderate physical activity			68.389**	<0.001
Yes	2,653 (48.36)	1,727 (40.00)		
No	2,833 (51.64)	2,591 (60.00)		
Light physical activity			74.685**	<0.001
Yes	4,638 (84.54)	3,356 (77.72)		
No	848 (15.46)	962(22.28)		
Smoking			68.562**	<0.001
Yes	2,651 (48.32)	1,725 (39.95)		
No	2,835 (51.68)	2,593 (60.05)		
Drinking			110.288**	<0.001
Yes	1,995 (36.37)	1,140 (26.40)		
No	3,491 (63.63)	3,178 (73.60)		
Total	5,486 (100.00)	4,318 (100.00)		

*Description *p*<0.05, **Description *p*<0.001.

**Table 3:** Multifactorial logistic regression analysis of independent factors on cognitive impairment.

Variables	OR	95%CI	*p*
Gender			
Male	1.00		
Female	1.54	1.35 ~ 1.77**	<0.001
Age			
60 ~ 64	1.00		
65 ~ 69	0.86	0.77 ~ 0.95*	0.005
70 ~ 74	0.89	0.78 ~ 1.01	0.061
≥75	1.51	1.33 ~ 1.73**	<0.001
Education level			
Illiterate	1.00		
Primary school and below	0.46	0.42 ~ 0.51**	<0.001
Middle school and above	0.62	0.54 ~ 0.70**	<0.001
Residence			
The center of city/town	1.00		
combined urban and rural areas	1.35	1.10 ~ 1.65*	0.004
Village	2.35	2.07 ~ 2.65**	<0.001
Special area	2.44	1.07 ~ 5.56*	0.034
Marital status			
Married	1.00		
Divorced/Widowed/Unmarried	1.40	1.25 ~ 1.57**	<0.001
Daytime napping			
0	1.00		
1 ~ 60 min	0.80	0.72 ~ 0.88**	<0.001
>60 min	1.07	0.95 ~ 1.20	0.282
Nighttime sleep duration			
≤5 h	1.00		
5 ~ 7 h	0.69	0.62 ~ 0.76**	<0.001
≥7 h	1.06	0.95 ~ 1.18	0.275
Social communication			
No	1.00		
Yes	0.69	0.64 ~ 0.76**	<0.001
Moderate physical activity			
No	1.00		
Yes	0.80	0.73 ~ 0.87**	<0.001
Light physical activity			
No	1.00		
Yes	0.84	0.75 ~ 0.94*	0.003
Smoking			
No	1.00		
Yes	1.13	1.00 ~ 1.29*	0.049
Drinking			
No	1.00		
Yes	0.87	0.79 ~ 0.97*	0.009

*Description *p*<0.05, **Description *p*<0.001.

## 4 Discussion

Cognitive impairment, as an early stage in the development of dementia, can be reversed to normal cognitive function in some patients (18). This stage of cognitive impairment also becomes the best window to intervene in its further development into dementia (19). The data analysis in the present study revealed that the overall prevalence of cognitive impairment in people over 60 was 44.04%, significantly higher than the 15.4% reported in previous studies (10). The analysis showed that different ways of determining cognitive impairment may lead to fluctuations in prevalence, and the Chinese version of the MMSE was used in this study as an assessment of cognitive function, which may have resulted in a higher positive rate of cognitive impairment due to the cultural bias of the MMSE (20) and the low response rate of older adults to the questionnaire content, which is one of the limitations of this study. In the future, a combination of cognitive measurement tools can be used to obtain more accurate and comprehensive data. Second, we included study participants who lived mainly in rural areas, where the overall educational level of the rural population is relatively low, and educational attainment has been shown to affect cognitive function (21).

Further analysis reveals that multiple factors, including demographic characteristics, health status, and lifestyle, influence the prevalence of cognitive impairment in older adults. Among them, aging-induced neurodegenerative changes are an essential cause of cognitive decline, and changes in the gray and white matter of the brain, reduction in the size and number of neurons, and impaired neurotransmitter performance can all lead to cognitive impairment (22).In this study, the percentage of cognitively impaired individuals in the female group was 50.8%, which was much higher than the 37.1% in the male group. This result corresponds to a cross-sectional study published in The Lancet, which showed that the prevalence of cognitive impairment in females was significantly higher than in males (23). The result is closely related to the physiological differences in the development of neurons in the early life of men and women and the influence of sex steroid hormones in adulthood (24).

In addition to the immutable factors of age and gender, the maintenance of cognitive functioning requires the support of individuals at multiple levels, including physical, psychological, and social. The present study reaffirms the protective effect of literacy on cognition, with higher literacy increasing an individual’s cognitive reserve and thus slowing the process of cognitive decline. In the Chicago Health and Aging Study, research has shown that education protects against cognitive decline and that the longer the duration of formal education, the lower the risk of neurodegenerative diseases (25). In addition, marital status is a significant factor in cognitive impairment, with divorced/widowed/unmarried status being a risk factor for cognitive impairment compared to married people, who are less likely to experience cognitive impairment because of the presence and support of their spouses. They are less likely to have negative emotions of loneliness and depression, thus reducing the incidence of cognitive impairment. This study also found that the prevalence of cognitive impairment among older people in rural areas and special regions was significantly higher than in towns. Analysis suggests that this may be because rural areas lag behind cities regarding economic conditions and education levels. At the same time, urbanization has brought about improvements in living conditions and higher income levels (26). However, since fewer people reside in special regions in the present study, the sample size was insufficient. Therefore, the prevalence of cognitive impairment fluctuated more with age when stratified by residence. Improving the older adult service system in rural and underdeveloped areas, strengthening cognitive screening, and providing health education are priorities for the future. For people with different levels of cognitive impairment, follow-up studies should explore individualized and precise interventions.

Lifestyle is also a necessary factor influencing cognition in old age. In this study, moderate daytime napping, nighttime sleep, and moderate drinking habits were associated with a lower risk of cognitive impairment. Sleep is an indispensable physiological process for maintaining brain health. Adequate sleep not only restores brain power but also contributes to memory consolidation and clearance of excess beta-amyloid and hyperphosphorylated tau proteins from the brain. Chronic sleep deprivation, on the other hand, can cause an inflammatory response that accelerates cognitive deterioration (27). The effects of alcohol consumption on cognition are more complex, and a dose–response meta-analysis of alcohol consumption has shown that light, moderate alcohol consumption reduces the risk of cognitive deficits when compared with non-drinkers (28). Bioactive substances contained in alcoholic beverages may play a protective role by attenuating oxidative damage to the organism (29). Excessive consumption of alcohol, on the other hand, can impair cognition. The dose–response relationship between alcohol intake and cognition needs to be explored in depth. Cigarette smoking is a recognized risk factor for cognitive decline. Nicotine contained in cigarettes can cause structural and neurochemical changes in the brain, leading to addiction, which is a coercive behavior with cognitive impairment characteristics (30). Changing these bad habits is essential for maintaining cognitive health in older people.

In addition, social participation and physical activity levels are closely related to cognitive function. The results of this study show that social participation and light to moderate physical activity have a protective effect on cognitive impairment. Maintaining a moderate level of socialization can help to obtain emotional support and reduce loneliness, and participating in group activities such as chess and card games and handicrafts can help to actively cope with mental tasks and strengthen cognitive abilities, and participating in more frequent social activities, especially educational activities, can have a better protective effect on cognitive functioning (31). In addition to benefiting cardiovascular function, physical activity can affect cognitive function through various mechanisms. For example, moderate- to high-intensity exercise activates the BDNF signaling pathway, promotes synaptic plasticity and neural regeneration, increases cerebral blood perfusion, and improves metabolism in cognitively relevant brain regions (32). Therefore, creating a favorable activity environment for older adults and encouraging community participation and physical activity is expected to slow down the process of cognitive decline.

This study has the following shortcomings: this study was based on cross-sectional data, and the determination of causality of cognitive impairment needs to be demonstrated in more intensive cohort studies and pilot studies, and the measurement of certain variables such as lifestyle and social participation relied on self-reporting by the study participants, and the data may be subject to bias. The Chinese version of the MMSE was used in this study to assess cognitive function, and although it was culturally adapted, it may have a certain degree of cultural bias, which may affect the accuracy of the prevalence of cognitive impairment. Therefore, future studies may consider the combined use of multiple cognitive screening scales, neuropsychological tests, clinical interviews, and other measures to obtain more comprehensive and objective cognitive functioning assessment results. In addition, the sample data for this study is still small relative to the overall population of the country, and larger sample sizes will be needed in the future to illustrate the overall cognitive profile of older adults in the country.

## 5 Conclusion

The prevalence of cognitive impairment in older people aged 60 years and above in China is at a high level, with advanced age, female gender, and low literacy being the main risk factors, while suggesting the protective effects of social participation, physical activity, and healthy lifestyle on cognitive function. In the future, community-based and family-based cognitive screening and comprehensive intervention systems for older people should be improved to provide practical guidance for addressing the challenges of cognitive impairment in the context of aging.

## Data Availability

The original contributions presented in the study are included in the article/supplementary material, further inquiries can be directed to the corresponding author.
